# Effects of Environmental Temperature and Humidity on the Geometry and Strength of Polycarbonate Specimens Prepared by Fused Filament Fabrication

**DOI:** 10.3390/ma13194414

**Published:** 2020-10-03

**Authors:** Lichen Fang, Yishu Yan, Ojaswi Agarwal, Shengyu Yao, Jonathan E. Seppala, Sung Hoon Kang

**Affiliations:** 1 Department of Mechanical Engineering and Hopkins Extreme Materials Institute, Johns Hopkins University, Baltimore, MD 21218, USA; lichenfang@jhu.edu (L.F.); yishuyan@berkeley.edu (Y.Y.); oagarwa1@jhu.edu (O.A.); syao15@jhu.edu (S.Y.); 2 Materials Science and Engineering Division, National Institute of Standards and Technology, Gaithersburg, MD 20899, USA

**Keywords:** fused filament fabrication, environmental temperature, humidity, X-ray micro-computed tomography, porosity, mechanical properties

## Abstract

It is widely known that the printing quality of fused filament fabrication (FFF) is heavily affected by environmental temperature and humidity, taking the form of warping and porosity. However, there is little understanding about the quantitative relations between environmental conditions, geometry, and the mechanical properties of printed parts. In this study, we systematically investigated those relations using bisphenol A polycarbonate as a model material system. For the environmental temperature, an in-situ infrared imaging analysis revealed the presence of an up to 5.4 °C/mm thermal gradient when printing using an open-chamber printer and a heated build plate. For the environmental humidity, an analysis of X-ray micro-computed tomography (micro-CT) scans showed an up to 11.7% porosity that was brought by polymer water content absorbed from environmental moisture. Meanwhile, tensile tests showed a mechanical performance loss associated with those defects, but, surprisingly, the transverse direction ductility had the potential to increase at a higher porosity. Furthermore, the experimental results were combined with analytical and parametrical studies to elucidate quantitative relations between environmental conditions and printing quality. Based on the results, quantitative guidelines for the estimation of printing quality based on environmental conditions are provided that would also help users to obtain desired printing results with a better understanding of the effects of environmental conditions.

## 1. Introduction

Fused filament fabrication (FFF) is one of the most popular additive manufacturing methods. It allows users with minimum training to easily convert their digital designs into real items at a low cost. Hence, it is widely applied in highly customized parts and prototyping [1]. However, compared to other fabrication methods, relatively inferior mechanical properties and commonly appearing printing defects limit FFF for more applications in industrialized productions [2,3]. To overcome this issue, scientists and engineers have been devoted to improving the FFF process and printing quality by optimizing printing parameters [4,5,6]. Nevertheless, environmental factors, such as chamber/build plate temperature and humidity [7], have long been insufficiently studied even though it was reported that they could significantly impact the printing quality, including by introducing geometrical defects like warping and porosity and by reducing mechanical performance parameters like strength [8,9].

To mitigate temperature-induced defects, a low cost and straightforward solution is adding a heated build plate to keep the specimen warm during printing. However, it was shown that a heated build plate could not provide a uniform temperature field across the entire building area, so thermal-induced defects still existed [10]. The more expensive but effective solution is controlling the temperature of the printing chamber, and there have been multiple numerical studies focusing on its effects on melt polymer flow [11], interfacial bonding [12], heat transfer [13], specimen distortion [14,15], etc. However, since most open-source FFF printers do not have temperature-controlled chambers, there have been much fewer experimental efforts reported. Sun et al. built a chamber for printing polyether ether ketone (PEEK), which requires a high environmental temperature to print, and they found a significant improvement in specimen strength [16]. Spoerk et al. printed polypropylene (PP) filled with glass spheres, which showed better annealing quality and dimensional accuracy with an elevated chamber temperature [17,18]. Carneiro et al. found that a 20 °C increase in environmental temperature reduced the structural porosity of printed acrylonitrile butadiene styrene (ABS) by 50% [19]. Armillotta et al. studied the specimen warpage with both analytical and experimental approaches, and they reported that a high chamber temperature close to the glass transition temperature significantly reduced the warpage [20].

Environmental humidity is another primary source of printing defects in FFF, though it is highly varied from material to material due to the different water absorption properties of various polymers [21]. For the polycarbonate (PC) that we are interested in, there were some water absorption studies decades ago that investigated physical property changes [22] and influence to injection molding applications [23]. Ito et al. found PC could absorb up to 0.2% by mass of water within 3 h under 24.5 °C and 88% relative humidity (RH) [24]. Multiple studies have demonstrated that the mechanical properties of PC are reduced upon water absorption. The primary mechanism behind this property reduction has been attributed to microstructural defects because the absorbed water clusters can lead to the formation of microcracks [23,25,26].

However, water absorption is closely related to material geometry (cylindrical filaments in our case), and the FFF process is also different from injection molding due its heating time, material quantity, and seal of the mold. Thus, a study specifically designed for FFF is required. There have been very few papers published in this area. Halidi et al. studied the moisture sorption effects on ABS filaments and confirmed it would not cause nozzle clogging [27]. Kim et al. studied the water absorption of printed ABS specimens and their corresponding property changes. Humidity was introduced together with a higher temperature, and they found that the printed parts had significant aging effects and the strength was reduced when storing in hot and humid conditions [28]. Valerga et al. studied the effects of humidity on polylactic acid (PLA) printing, and they found the material could degrade during storage and created bubbles when printing, thus reducing the mechanical strength of printed parts [29].

Overall, researchers have noticed the negative effects brought by environmental conditions, but more thorough and systematic studies are needed to quantitatively understand those influences on the printing process. For this paper, we investigated those issues step by step. For temperature effects, we first measured the temperature field using an infrared camera and obtained the thermal gradients within specimens printed under different environmental temperatures. Then, from the thermal gradients, we predicted the warpages and compared them with experimental measurements. Finally, we performed tests to capture the mechanical property changes. For humidity effects, we started with a water absorption test to quantify how much water printed materials could absorb under different levels of humidity. With the water content, we estimated the corresponding porosity, which was compared with experimental results obtained from micro-computed tomography (micro-CT) scans. Eventually, uniaxial tensile test results were analyzed along with pore size data to understand processing–microstructure–property relations.

## 2. Materials and Methods 

### 2.1. Fabrication

**Figure 1:**
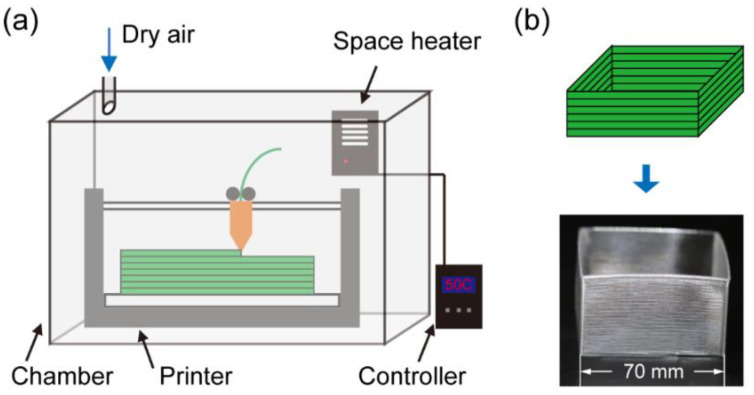
(**a**) Schematic of the printer and the environmental control system; (**b**) the illustration and picture of the printed hollow box geometry.

A LulzBot TAZ 6 printer (Fargo Additive Manufacturing Equipment 3D, LLC, Fargo, ND, USA) with 0.5 mm nozzle diameter was used as the main equipment for fabrication, around which a low-cost environmental control system was built. As Figure 1a shows, the printer was enclosed with an acrylic chamber (Printed Solid, Inc., Newark, NJ, USA). A hole was cut on the top surface to connect to compressed dry air, which was pumped in during printing to keep the environmental humidity below 10% relative humidity (RH). A space heater (STEGO, Inc., Kennesaw, GA, USA) was mounted inside the chamber and connected to a temperature controller that could vary the environmental temperature from room temperature (20 °C) to 90 °C. For the filament feedstock, bisphenol-A-polycarbonate was used as the model material system. The original mechanical properties of the 2.85 mm PC filaments (Ultimaker B.V., Utrecht, The Netherlands) were measured for comparison (see Figure S1 and Table S1 for details). To keep the initial conditions constant, all filaments were dried for 1 h in a vacuum oven set at 100 °C. Meanwhile, PC’s zero-shear viscosity was obtained by performing a 120–280 °C temperature sweep measurement using a rheometer (Anton Paar GmbH, Graz, Austria).

To study the effects of varying environmental conditions, temperature and humidity were varied separately, i.e., the environmental humidity was kept below 10% RH when studying temperature effects and the temperature was set at 22 °C when studying humidity effects. Afterward, the corresponding physical and mechanical effects were investigated. For environmental temperature, four different values (30 °C, 50 °C, 70 °C, and 90 °C) were tested—the entire chamber was heated and kept around a target temperature during the printing process. The temperature fluctuation was around ±5 °C, since the acrylic chamber had limited thermal insulation. For the effects of PC water absorption under different levels of humidity, a lab balance attached with a Cellkraft P-2 humidifier (Cellkraft AB, Stockholm, Sweden) was used (see Figure S2a for details). The environmental humidity could be controlled with a ±1% RH fluctuation. Dried PC filaments were then exposed to four different humidity levels (10% RH, 30% RH, 50% RH, and 70% RH) for 24 h to study the water absorption rate, and the resulting filaments with different water contents (0% by mass, 0.05% by mass, 0.1% by mass, and 0.15% by mass) were printed and tested. Despite the varied environmental conditions, all other printing parameters were kept same, including layer height (0.3 mm), nozzle temperature (280 °C), print speed (10 mm/s), and build plate temperature (115 °C).

The bonding between two stacked fiber tracks is a critical part that determines the properties of FFF printed parts. To study how the bonding property changes with environmental conditions, we created a 70 mm by 70 mm by 50 mm hollow box geometry, as Figure 1b shows. Each side of the box was a single-track wide wall made with multiple tracks printing one over another. The width of the wall was set to be equal to the nozzle diameter of 0.5 mm. After fabrication, the specimens were cut to smaller pieces for either the micro-CT scan or mechanical tensile tests.

### 2.2. Characterizations

**Figure 2:**
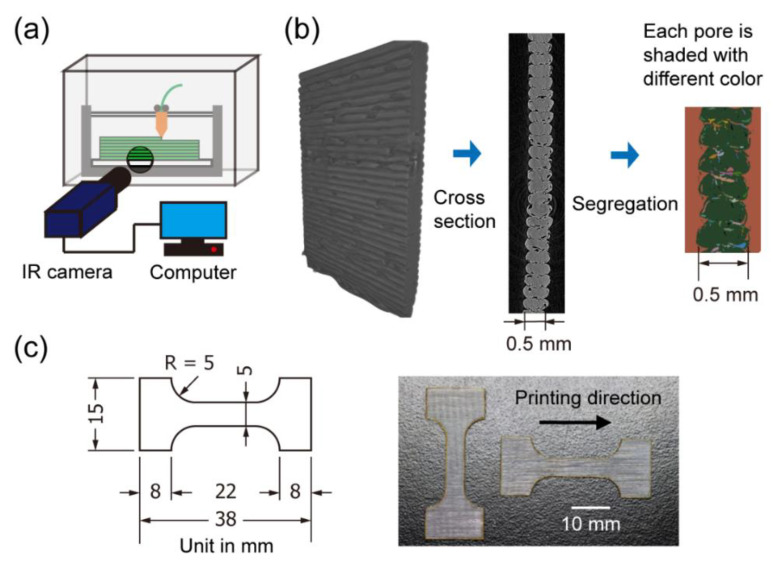
(**a**) Schematic of the infrared thermography system; (**b**) illustration of the void segregation of scanned specimens; (**c**) drawing and pictures of the dog bone tensile specimens.

Three characterization techniques were applied to measure the property changes upon varying environmental conditions. First, for the temperature side, infrared (IR) thermography was used to evaluate the temperature field of the specimen during printing. As Figure 2a shows, an IR camera (FLIR a6701sc, FLIR Systems, Inc., Wilsonville, OR, USA) with a 50 mm F/2.5 lens was mounted to face a round opening in the front panel of the printer chamber. The opening was drilled to allow for infrared light transmission because acrylic is not transparent to infrared. A calibration of the PC emission spectrum was performed, as shown in Figure S3 [30]. The measured photon counts were later imported into MATLAB for converting to the temperature field and performing further analysis. During printing, we found the extruded material temperature could be 15 °C lower than the nozzle temperature set at 280 °C. However, IR thermography could not measure the material temperature when it was inside the nozzle, and the material cooled down rapidly after leaving the nozzle. Thus, the real material temperature could be between 265 °C and 280 °C. After printing, pictures of the specimens were taken using a Canon EOS 80d digital single-lens reflex (DSLR) (Canon Inc., Tokyo, Japan) with a 100 mm F/2.8 ultra sonic motor (USM) stabilized macro lens. The images had a resolution of 15 μm/pixel and were later used to quantify the warping deformation associated with the thermal gradient.

To gain more understanding on the humidity effect, the as-printed specimens were scanned by Bruker Skyscan 1172 micro-CT (Bruker Corporation, Billerica, USA) with a 4.87 μm resolution. All pores larger than 10 μm could be identified and segregated (smaller pores could not be detected) using the post-processing software package Dragonfly (Dragonfly Software, GA, USA). As Figure 2b shows, each pore was separately labeled using the watershed transformation algorithm, and then statistics of the pore size distribution and overall porosity could be obtained.

Finally, tensile tests were conducted on all printed specimens using an Instron E1000 mechanical testing system (Instron, Norwood, USA). Following the ASTM D1708 standard, the specimens were laser cut into a dog bone geometry with a 12 mm by 5 mm gauge area, as Figure 2c shows. The tests were performed quasi-statically with 0.01 mm/s testing speed. The specimens were tested both along and perpendicular to the printing direction to investigate the anisotropy. The resulting force–displacement curves were then normalized to stress–strain curves for more general comparisons. The strain was measured by optical monitoring (see Figure S4 for details), and the stress was calculated via forces divided by CT-measured average cross-section areas (see Figure S5 for details).

To confirm the statistical significancy, all warping deflections and mechanical testing results were examined with analysis of variance (ANOVA) using the MATLAB statistics toolbox. This analysis could compare the mean values between groups of samples and determine whether they had the same mean values. 

## 3. Results and Discussions

### 3.1. Effects of Varying Environmental Temperature

In this subsection, the relations among environmental temperature, specimen thermal gradient, warping defects, and mechanical properties are investigated and discussed. An analytical estimation of the warping deformation is also proposed and examined.

#### 3.1.1. Characterizations of Temperature Fields within Specimens

**Figure 3:**
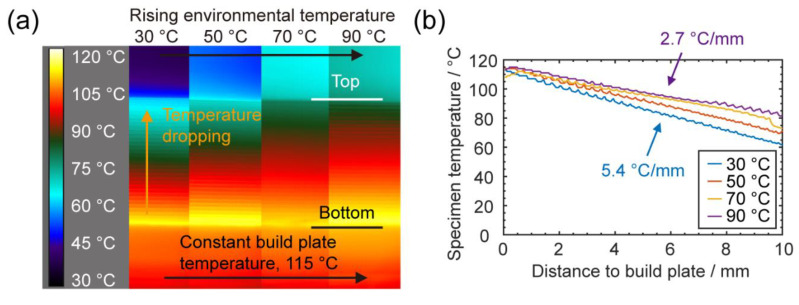
(**a**) False color infrared images of specimens being printed under different environmental temperatures; (**b**) the plot of specimen temperatures at different heights.

Using the infrared camera, the pseudo-color thermography images of four specimens were plotted and labeled in Figure 3a, representing the thermal profiles right after printing under four different environmental temperatures. Within the pictures, the bottom part is the build plate, which has a constant temperature; the top part is the far-side acrylic chamber background (out of focus), which represents the rising environmental temperature; and in the middle is the specimen being printed, which has a layer-by-layer structure. It should be noted that the bright and dark strips in specimens do not indicate that there are quite different temperatures. They are mostly due to the differences in the surface normal—if the surface was facing the camera, the sensor received maximum radiation and had the correct temperature reading; otherwise at the connecting point between two layers, the surface normal was not pointing toward the lens and the infrared emission received by the lens was smaller so the temperature reading was lower. Due to similar reasons, we also saw that the build plate had a lower temperature reading than its set value.

In the images, it is obvious that the thermal gradient from bottom to top of the specimen changed significantly with the change in the environmental temperature. The temperature gradients are quantified and plotted in Figure 3b, from which we can see the specimen temperature decreasing with the distance from the build plate. At 30 °C, a 5.4 °C/mm thermal gradient was observed, while the thermal gradient was reduced to 2.7 °C/mm when the environmental temperature increased to 90 °C.

#### 3.1.2. Warping Defect Predictions and Measurements

**Figure 4:**
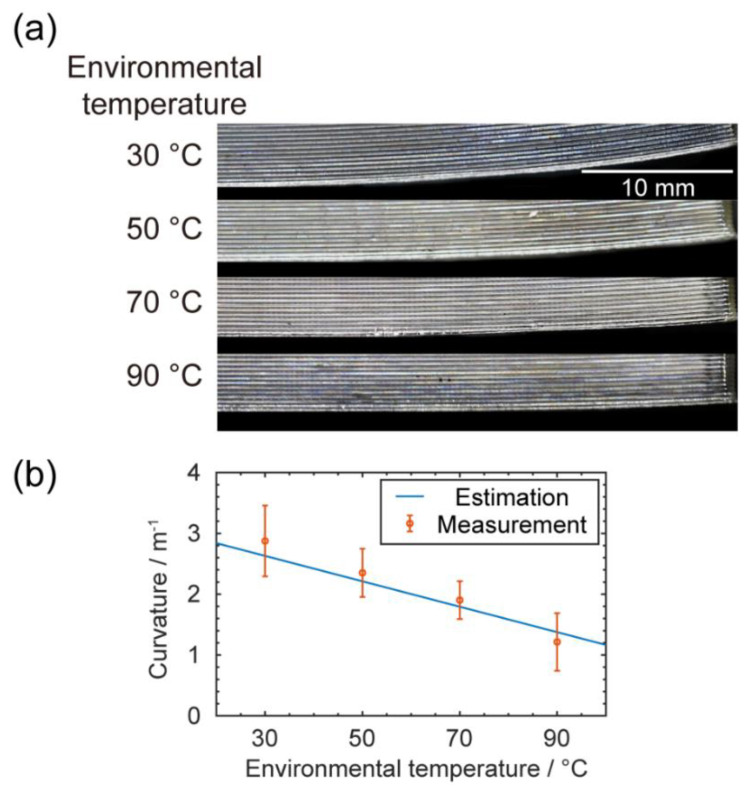
(**a**) Pictures of specimens with warping deflections; (**b**) the plot of predicted deflections and measurements of deflections with respect to the changing environmental temperatures. The error bars are from standard deviations of data obtained from five measurements.

With the thermal gradient, strain mismatches were brought by the different thermal expansions in different layers, causing warping defects. As known from polymer theory, PC is highly viscous before cooling down to the glass transition temperature (see Figure S6 for measurements); after passing that point, any further thermal shrinkage will create corresponding elastic thermal stress [20]. From previous studies, we could only consider elastic and plastic deformations in PC’s glassy state, while the viscosity was extremely high (>10^21^ MPa·s at room temperature) [31]. At higher layers, which were cooler, there was a larger temperature difference between specimen temperature and glass transition temperature, so the PC shrunk more than in the lower and warmer layers. As a result, the entire printed specimen tended to bend upward. This deflection was mitigated with a higher environmental temperature that generated a smaller thermal gradient, as Figure 4a shows.

Given the observed thermal gradient, it was possible to analytically predict the corresponding warpage. In our case, the maximum thermal strain was smaller than 2%, which was within the linear region of the stress–strain curve (Figure S1); thus, no plasticity was involved. For a slender beam without external constraint, the warping-induced curvature could be estimated as:

(1) $$\kappa =\alpha \left(T\right)\frac{(T_{g}-T_{\mathrm{top}})-\left(T_{g}-T_{\mathrm{bottom}}\right)}{H}=\;\alpha \left(T\right)\frac{\mathrm{dT}}{\mathrm{dz}}$$

where α(T) is the coefficient of thermal expansion (see Figure S7 for measurements), L is the beam length (considering the symmetry, we used 35 mm here), H is the beam height, T_g_ is the glass transition temperature, and T_top_ and T_bottom_ are the temperatures at top and bottom of the specimen, respectively. The temperature/height term is equal to the thermal gradient along height/Z direction, marked as dT/dz; the measured values were already given in Section 3.1.1.

However, since our specimen was not a slender beam, the shear deformation could not be ignored. Hence, we applied a correction based on Timoshenko beam theory, shown here as Equation (2) [32,33]:

(2) $$\kappa_{T}=\left(1+\frac{2\mathrm{EI}}{{\mathrm{KL}}^{2}\mathrm{AG}}\right)\alpha \left(T\right)\frac{\mathrm{dT}}{\mathrm{dz}}$$

where E is the elastic modulus, G is the shear modulus (E/G = 2.7 for PC), I is the second moment of area, A is the cross-section area, and K is the Timoshenko shear coefficient (equals to 5/6 for a rectangular section).

To evaluate the model, we compared the calculated curvatures with experimental measurements, as shown in Figure 4b. From analysis of variance (ANOVA), we found that the *p*-value was 2.16 × 10^−4^, indicating that the average curvature for each group was significantly different. The model could also capture this warping curvature decrease upon an increasing environmental temperature. The discrepancy, especially for the overestimation at 90 °C, could be attributed to the adhesion effects. The adhesion force between the build plate and the specimen could compensate for the bending moment induced by thermal stress, especially at higher temperatures when the thermal stress is smaller. Here in experiments, we tried to minimize the adhesion effects to fulfill the ‘no external constraint’ condition in our model. While in practice, the adhesion force could depend on multiple factors including material pair, build plate temperature, glues, and first layer geometry.

From this mechanism, there could be two ways to reduce thermal deflection. The first one is increasing the adhesion force between the build plate and printed specimen. However, a concern here is that the thermal stress still exists during printing, and if it is large enough, it may overcome the bonding force between layers and cause delamination. The second option is increasing the environmental temperature; from our observation, it continuously mitigated the warping defects.

#### 3.1.3. Impacts on Mechanical Properties

**Figure 5:**
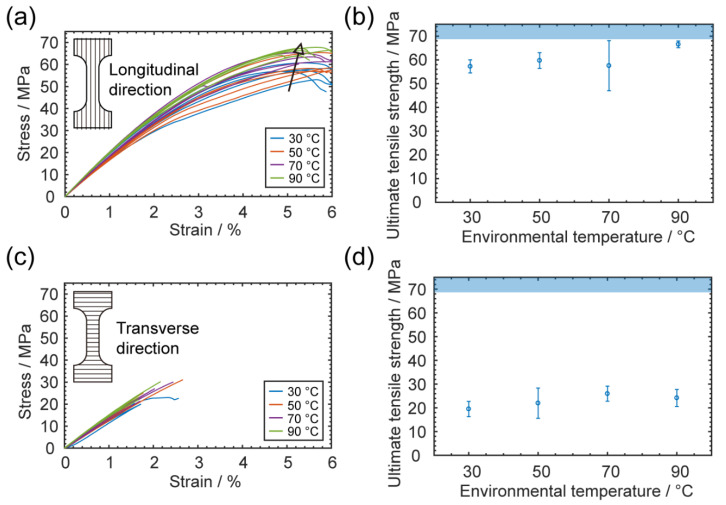
Mechanical effects of varying environmental temperature. (**a**) Stress–strain curves of tensile specimens tested longitudinally; (**b**) ultimate tensile strengths of longitudinal specimens printed with different environmental temperatures. The reference value of unprinted bulk polycarbonate (PC) is denoted as the pale blue shaded region; (**c**) stress–strain curves of tensile specimens tested transversely; (**d**) ultimate tensile strengths of transverse specimens printed with different environmental temperatures. The error bars are from standard deviations of data obtained from five measurements. The reference value of unprinted bulk PC is denoted as the pale blue shaded region.

Finally, the mechanical strengths of printed specimens were measured, and the stress–strain curves were plotted. For the longitudinal direction (stretching along the printing direction), the ductile mechanical responses were similar to those of bulk PC, as Figure 5a shows. In Figure 5b, the ultimate tensile strengths also show a slightly increasing trend with increasing environmental temperature; from ANOVA analysis, we found that the *p*-value was 0.024, indicating it was not a significant change. This increasing trend could have been due to the mitigation of geometrical defects, including warping and delamination. With less warping, the extrudates were better aligned to the testing direction, which provided higher strength. Eventually, by printing at 90 °C, the strength could approach that of the bulk material.

For the transverse direction (stretching perpendicular to the printing direction), the mechanical performance was significantly reduced and exhibited brittle behavior, suggesting a strong anisotropy, as Figure 5c shows. The earlier fracture of specimens was primarily attributed to the non-uniform cross-section geometry in the transverse direction; the bond between two layers was always narrower and hence easier to break. Thus, environmental temperatures played a less important role here. After comparing different environmental temperatures, we found that the *p*-value of strength in the transverse direction was 0.273, suggesting the change was not statistically significant, as shown in Figure 5d.

### 3.2. Effects of Varying Environmental Humidity

In this subsection, the relationship between environmental humidity, specimen porosity, and mechanical properties is investigated and discussed. An analytical estimation of the porosity is also proposed and examined.

#### 3.2.1. Water Absorption of PC

**Figure 6:**
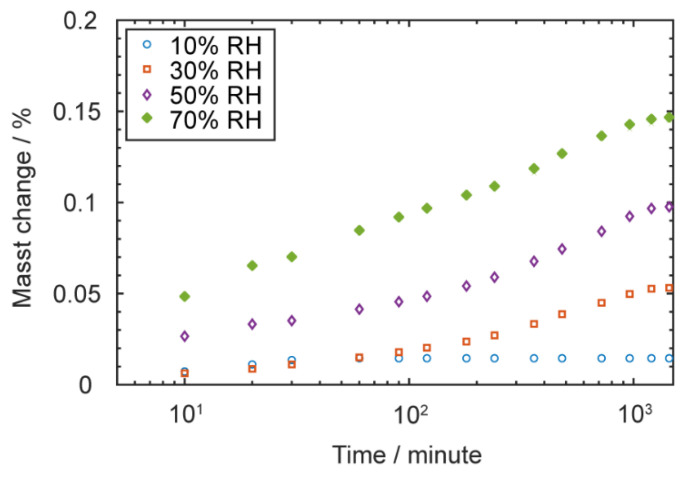
The mass change over time of PC filaments exposed to different humidity levels; specimens appeared saturated after 24 h.

To investigate the effects of environmental humidity, the first task was to understand how much water the PC could absorb. As described in Section 2.1, the mass change of 20 g of dried PC filaments was monitored for 24 h with exposure to five different humidity levels ranging from 10% RH to 90% RH with a 20% RH increment. After experiments, the filaments were re-dried and measured to make sure they returned to the original mass in order to confirm that the mass change was purely due to moisture absorption. The results are plotted in Figure 6, except for the results under 90% RH, in which we observed significant droplet condensation and mist in the lab balance chamber (see Figure S2b for details), so that measurement was discarded. 

All four curves showed very high water absorption rate at the beginning, and the rate gradually dropped with time until the curve reached a plateau. The measured saturation points were around 0.01%, 0.05%, 0.10%, and 0.15% by mass, respectively. The results were comparable with previously reported data [24]. When those filaments were printed, the stored water evaporated due to the heat and created undesirable pores within specimens. According to previous studies, the water diffusion depth in cylindrical filaments is proportional to the square root of time multiplied by diffusivity [34]. Thus, the interior core and exterior surface of filaments may have different water contents and result in different porosities and mechanical properties.

#### 3.2.2. Characterizations of Pore Defects

**Figure 7:**
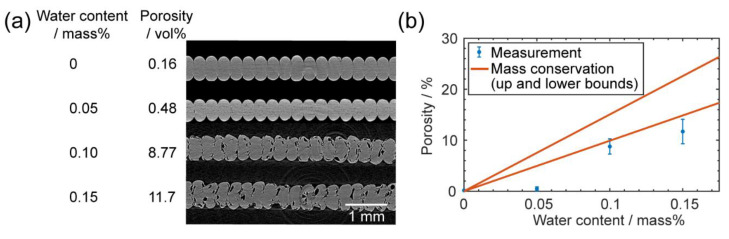
(**a**) The computed tomography (CT)-scanned cross sections of printed specimens using filaments with different water contents; (**b**) the comparison between measured porosity and predicted porosity using mass conservation.

We obtained several specimens by printing the filaments with different water contents. To characterize their porosity, micro-CT scans were conducted. Four representative scanned cross-sections of the specimens are plotted in Figure 7a. For printing with dried filaments, the porosity was as low as 0.16% by volume, while for high water content filament (0.15% by mass), the printed specimen had a porosity of up-to 11.7% by volume and the overall geometry was highly non-uniform.

If we assumed that all the water was vaporized and hence created pores inside the specimen, we could calculate the corresponding vapor volume from mass conservation. Similar to other gases, water vapor is not an ideal gas, and corrections need to be applied to the ideal gas law, as done here with Equation (3) [35]:

(3) $$\frac{P}{\rho \mathrm{RT}}=1+B\rho +{C\rho }^{2}+\cdots$$

where P is the pressure inside the nozzle; ρ is the molar density, which is correlated with both vapor mass and volume; R is the gas constant of 8.314 J/(K·mol); and T is the material temperature, which in principle shall be equal to the nozzle temperature of 280 °C (553 K) but in practice it could be lower considering thermal conduction takes time [36]. From our IR observations, the material temperature could have been 15 °C lower than nozzle temperature after coming out of the nozzle. This served as the lower bound of the material temperature since IR thermography could not measure the temperature inside the nozzle and the material cooled down rapidly in ambient environments. Thus, we made another calculation based on the temperature of 265 °C (538 K). Finally, B and C in Equation (3) are the virial coefficients that characterize the interaction potential between particles (B for two particles, C for three, etc.) and that provide systematic corrections to the ideal gas law. In practice, the higher-order virial coefficients (C and beyond) are often ignored because interactions between three and more molecules are less likely to happen in the gas phase [37]. However, the water vapor’s second virial coefficient B has been extensively studied and could be estimated by a fitted equation as Equation (4) [38]:

(4) $$\frac{B\left(T\right)}{B^{0}}=\overset{4}{\underset{i=1}{\sum }}a_{i}*{\left(T^{*}\right)}^{b_{i}}$$

where B_0_ was measured to be 1000 cm^3^/mol, T^*^ was T/100K, a_1_ was 0.34404, b_1_ was −0.5, a_2_ was −0.75826, b_2_ was −0.8, a_3_ was −24.219, b_3_ was −3.35, a_4_ was −3978.2, b_4_ was −8.3, and the calculated B at 553 K was −128.17 cm^3^/mol and −138.73 cm^3^/mol at 538 K.

The final unknown parameter is the internal pressure, which is hard to measure. A previous study reported an estimation approach derived from the Navier–Stokes equation, shown here as Equation (5) [39]:

(5) $$P_{\mathrm{nozzle}\_\mathrm{approx}}=\;\frac{12\mu \mathrm{qD}}{h_{l}^{3}}-\frac{6{\mu v}_{p}D}{h_{l}^{2}}$$

where the viscosity μ was measured to be 249.88 Pa·s for our PC under 280 °C and 354.83 Pa·s under 265 °C using an Anton Paar MCR 302 rheometer; q is the volumetric flow rate normalized by a unit length in the Y direction, which was 36.67 mm^2^/s for our printing parameters; D is the nozzle diameter of 0.5 mm; v_p_ is the print speed of 10 mm/s; h_l_ is the layer height of 0.3 mm; and, finally, the pressure was estimated to be 1.95 MPa at 280 °C and 2.77 MPa at 265 °C.

From Equations (3)–(5), we calculated the water vapor’s molar density (ρ) to be 450.10 mol/m^3^ at 280 °C and 684.23 mol/m^3^ at 265 °C. Then, the porosity was estimated as:

(6) $$\mathrm{Porosity}\;=\;\mathrm{water}\;\mathrm{content}\;{*\rho }_{\mathrm{PC}}/{\rho M}_{\mathrm{water}}$$

where the density of PC is 1.22 g/cm^3^ and M_water_ is the molar mass of water as 18 g/mol. Substituting different water contents into Equation (6) we could get the estimation of the porosity. A comparison between this mass-conservation model and measured values is plotted in Figure 7b, from which we can see that the model’s values were significantly higher than the measured values. This could have been due to two primary reasons; the first one was the water vapor may have escaped from the specimens, especially for water distributed near the exterior surfaces of the filaments; the second reason was the limitation of our measurement capability—our micro-CT scans ran at 4.87 μm/pixel, meaning all the pores smaller than that threshold remained undetected, even though they could have contributed to the overall porosity.

**Figure 8:**
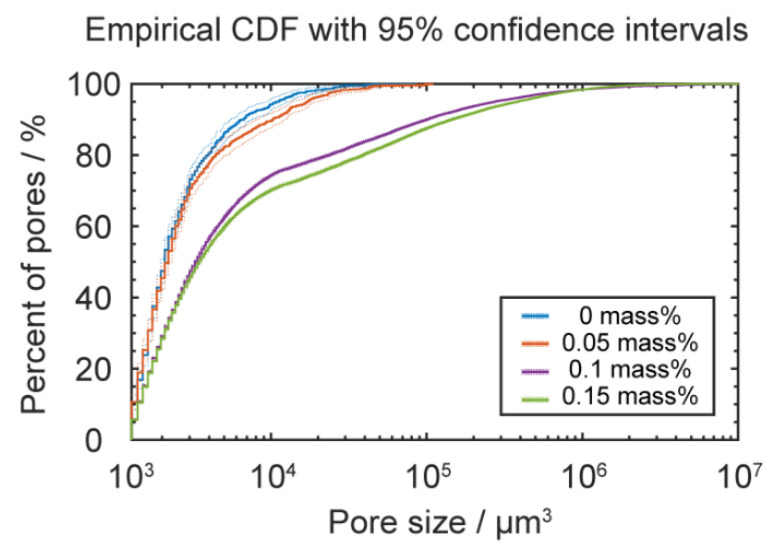
Empirical cumulative distribution functions (CDF) for the pore sizes of printed specimens using filaments with different water contents; dotted lines indicate the upper and lower bounds of 95% confidence intervals.

To further quantify the size distribution of those pores, the Dragonfly software package was used to segregate each pore and statistically analyze the pore size distribution. The results are shown in Figure 8, which illustrates the empirical cumulative distribution function (CDF) [40] for the pore sizes. We found that higher water content led to higher percentages of large pores, and the volume of the largest pores also increased with the water content. As a result, according to fracture mechanics, samples with larger pores should have had a lower mechanical strength because the pores served as initiation sites and helped propagate cracks.

#### 3.2.3. Impacts on Mechanical Properties

Finally, we investigated the mechanical performance changes with pore defects. Previous studies have shown that injection-molded PC’s mechanical properties drop upon water absorption, mainly due to two reasons: one is that absorbed water clusters can lead to the formation of microcracks [23,25,26], and the other is the molecular weight reduction upon hydrolysis [41]. For our case, the printed materials were extruded from the hot nozzle and then kept on the 115 °C build plate for hours so that the specimen was expected to have minimal water content after printing. This was further confirmed by additional drying experiments in which the printed specimens were measured to have the same mass before and after drying. Even without any water content, the pore defects remaining inside the specimen from the water absorbed by the filament could still impact the mechanical properties; this was accounted for as a geometrical factor and is discussed below. The hydrolysis reaction is a much slower process that takes months to show a significant difference [41], so it did not need to be accounted for in this study.

**Figure 9:**
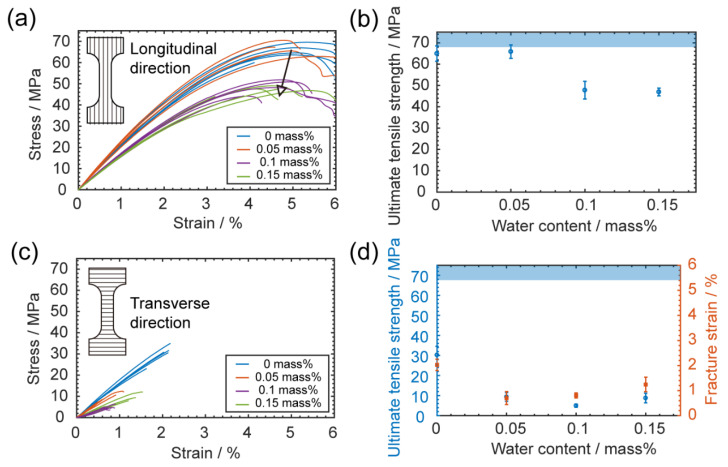
Mechanical effects of varying water content. (**a**) Stress–strain curves of tensile specimens tested longitudinally, and the arrow indicates the dropping strength with higher water content; (**b**) ultimate tensile strengths of longitudinal specimens printed with different water contents, the reference value of the unprinted bulk PC is denoted as the pale blue shaded region; (**c**) stress–strain curves of tensile specimens tested transversely; (**d**) ultimate tensile strengths and fracture strains of transverse specimens printed with different water contents. The error bars are from standard deviations of data obtained from five measurements. The reference value of the unprinted bulk PC is denoted as the pale blue shaded region.

For the longitudinal direction (stretching along the printing direction), the mechanical behavior was still ductile, as Figure 9a shows. However, there was a significant loss in ultimate tensile strength when printing with a higher water content (*p*-value is 6.91 × 10^−5^ under ANOVA analysis). As Figure 9b shows, the strength decreased by around 30% compared to printing with dry filament, for which the strength was close to that of a bulk PC. This loss could be attributed to the porosity. The randomly distributed pores reduced the effective area of the cross-section and created a stress concentration that greatly weakened the specimens.

**Figure 10:**
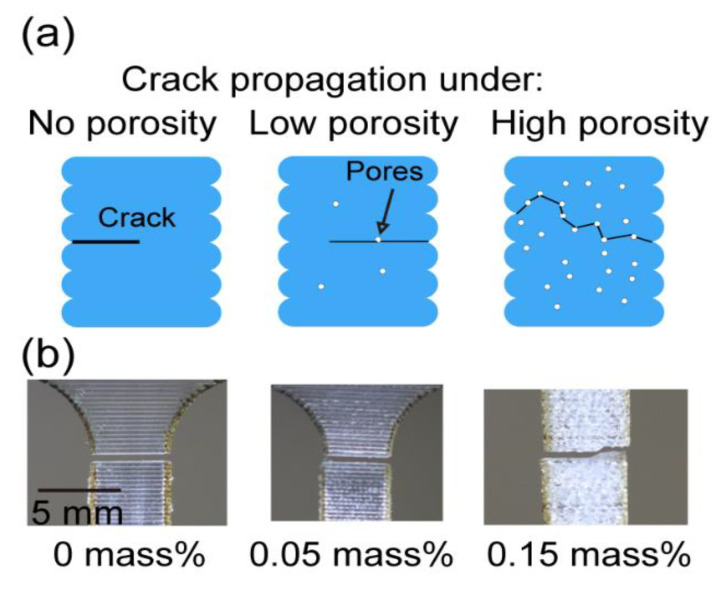
(**a**) Illustrations of crack formations with different porosities; (**b**) images of cracks.

For the transverse direction (elongated perpendicular to the printing direction), the mechanical performance was again reduced and the deformation behavior became brittle, meaning the ultimate tensile strength was also the fracture strength, as Figure 9c shows. Here, we observed a very interesting trend of mechanical property change (*p*-value is 5.26 × 10^−10^): both fracture strength and strain initially decreased but later increased with the increase in water content, as shown in Figure 9d. This phenomenon only happened in the transverse direction, suggesting that it could be a combined effect of the printed geometry and pore defects. As the illustrations in Figure 10a show, when there was no porosity, the weld point between the two layers could initiate the crack and the crack propagated along the relatively narrow bond; when some porosity was introduced, the extra pores could help the crack initiation and propagation, leading to a lower fracture strength. However, when the porosity was high, the randomly distributed pores may have deviated the crack and let it propagate along a longer path, which might have introduced some extra ductility to the specimens. Evidence for this hypothesis is shown in the pictures in Figure 10b, from which we can see for no water content, the crack was exactly along the bond between two layers. Meanwhile, for the higher water content, the crack deviated from the bond and propagated across multiple layers.

As a summary, the porosity brought by moisture absorption could significantly impact both the geometry and mechanical performance of printed specimens. The pores not only made the product rougher but also decreased the uniformity of the printing pattern. For the mechanical side, the strengths decreased in both the longitudinal and transverse directions with water content. Though some extra ductility could be introduced at a very high water content, the mechanical performance was still worse than that of printing with dried filaments.

Combining the mechanical tests with the water absorption test results shown in Section 3.2.1 while avoiding water in the print, we found that it was not sufficient to only dry filaments beforehand. However, it was also important to minimize the moisture absorption during printing. The absorption rate of water into PC is very fast at the beginning. For example, if less than 0.05% by mass water content is desired, printing could either be performed under 30% RH with dry filaments—as long as the printing does not take a full day; otherwise, a dry environment is also required because PC can absorb 0.05% by mass of water in less than 2 h at > 50% RH humidity.

## 4. Conclusions and Outlook

In this study, we systematically investigated the geometrical and mechanical effects of varying environmental temperature and humidity during the FFF process using a combination of analytical and experimental approaches. Regarding temperature, the temperature gradient within specimens was quantified, and the resulting warping defects were estimated and measured. The model and experiments showed that increasing the environmental temperature from 30 °C to 90 °C could mitigate the warping defects by a 50% reduction in curvature and could improve mechanical performance along the filament printing direction. Meanwhile, binding the specimen to the build plate is a worse choice (compared to increasing environmental temperature) because delamination may still happen.

Regarding humidity, the water absorption rate and saturation points of PC filaments were measured under different environmental humidity levels. The saturated filaments were then printed and characterized. While the water-generated porosity was less than estimated, it still showed a significant impact on the specimen’s mechanical performance. Compared to printing with dried filaments, the specimens made of filaments with a water content had an inferior strength in both the longitudinal (up to a 30% reduction) and transverse directions (up to a 70% reduction). However, compared to specimens with a low water content (>0% by mass and <0.10% by mass), up to a 50% increase in ductility was observed in the transverse direction for the higher water content (>0.10% by mass) specimens. This increase in ductility could have been due to the combined effect of high porosity and non-uniform cross-section geometry: the crack propagated beyond the bond plane and the long path in return gave more plastic dissipation. Overall, we recommend drying filaments before printing and to keep the printing environment dry and heated, especially for long jobs, for minimum geometric warping and good mechanical performance.

Besides practical recommendations to printer users, we envision our study opens new approaches to better estimate printing quality based on environmental conditions, which has been insufficiently studied before. Future research directions include both expanding to large scale samples and exploring other materials. For large scale samples, other parameters like the raster angle, infill density, and tool path will play important roles. Thus, the thermomechanical behaviors and the porosity-induced mechanical change will be more complex for prediction. For materials other than PC, different properties like crystallinity may need to be considered. To fully account for all those factors, a multi-physics, multi-scale numerical model that considers multiple aspects including solid mechanics, polymer dynamics, and molecular and chain dynamics is needed in the future. We hope our study could provide solid experimental foundations for such numerical studies.

## Supplementary Materials

The following are available online at https://www.mdpi.com/1996-1944/13/19/4414/s1: Figure S1: Uniaxial tension plot of Ultimaker filaments before and after printing; Table S1: Tabulated values of uniaxial tension specimens; Figure S2: Setup of the water absorption test; Figure S3: Calibration of PC’s infrared emission; Figure S4: Measurements of strain within gauge area; Figure S5: Measurements of cross-section area with micro-CT; Figure S6: Measurements of PC’s glass transition temperature; and Figure S7: Measurements of PC’s coefficient of thermal expansion.

## Disclaimers

Official contribution of the National Institute of Standards and Technology; not subject to copyright in the United States. Certain commercial equipment, instruments, or materials are identified in this paper in order to adequately specify the experimental procedure. Such identification is not intended to imply recommendation or endorsement by the National Institute of Standards and Technology, nor is it intended to imply that the materials or equipment identified are necessarily the best available for the purpose.
