# Supplementary figures and images for: Effects of Environmental Temperature and Humidity on the Geometry and Strength of Polycarbonate Specimens Prepared by Fused Filament Fabrication
Source: Materials (Basel). 2020 Oct 3;13(19):4414. doi: 10.3390/ma13194414 (PMC7579282; doi:10.3390/ma13194414)

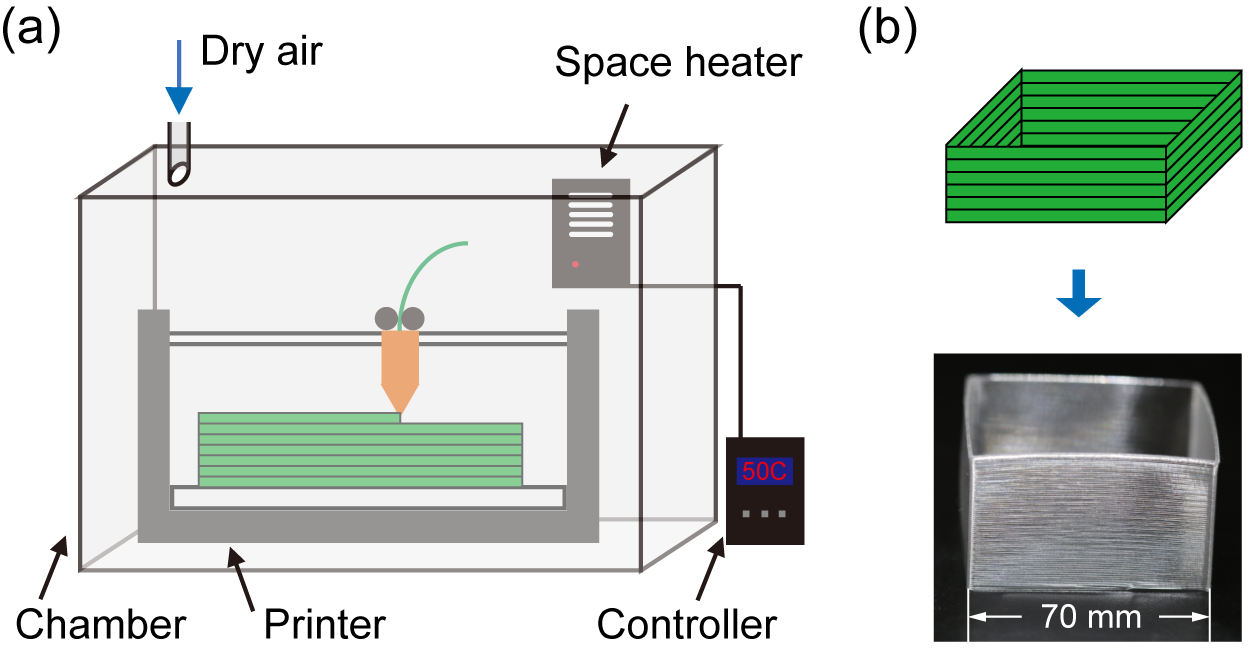

Supplement: Supplementary file 1 [file materials-13-04414-s001.zip › Fig1.tif]

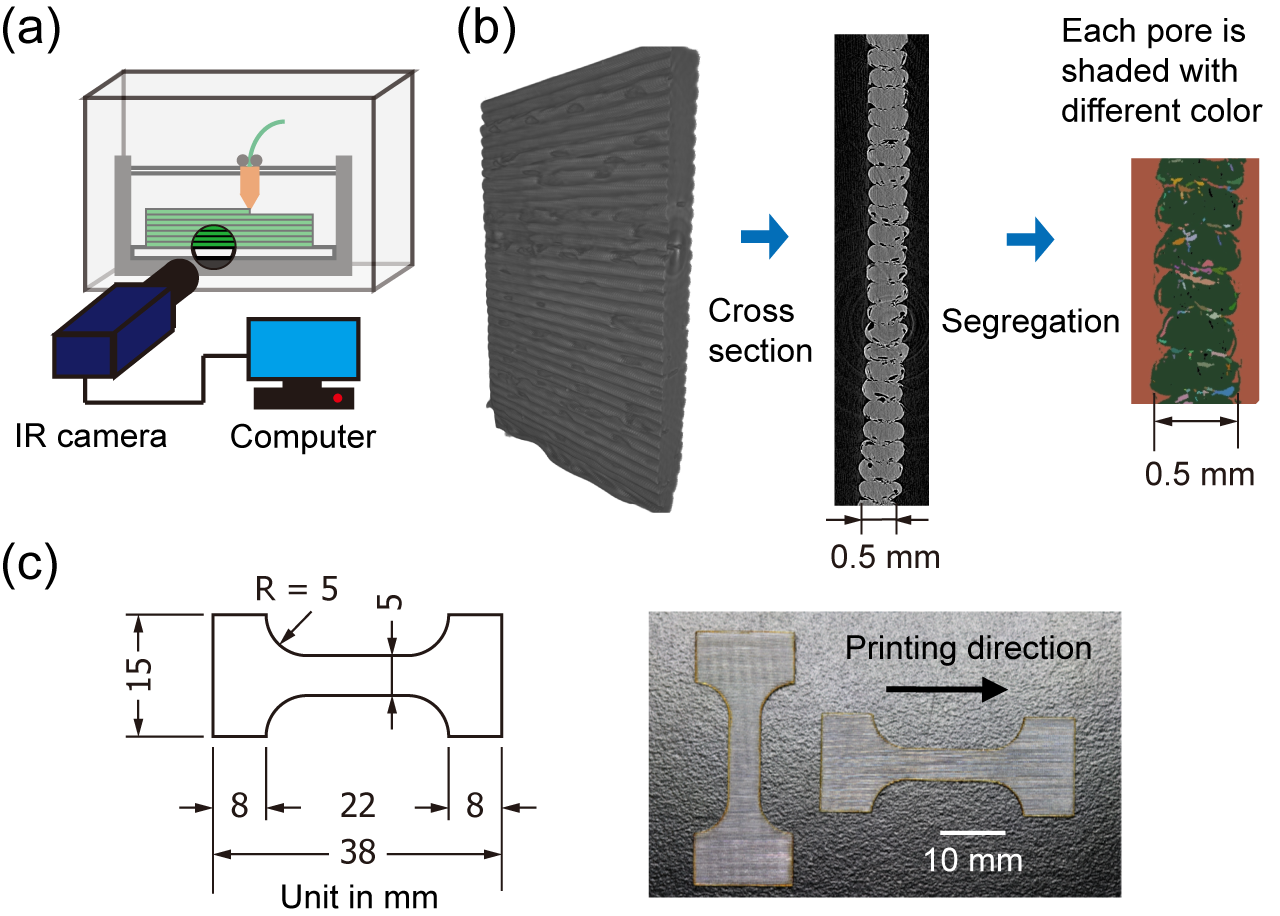

Supplement: Supplementary file 1 [file materials-13-04414-s001.zip › Fig2.tif]

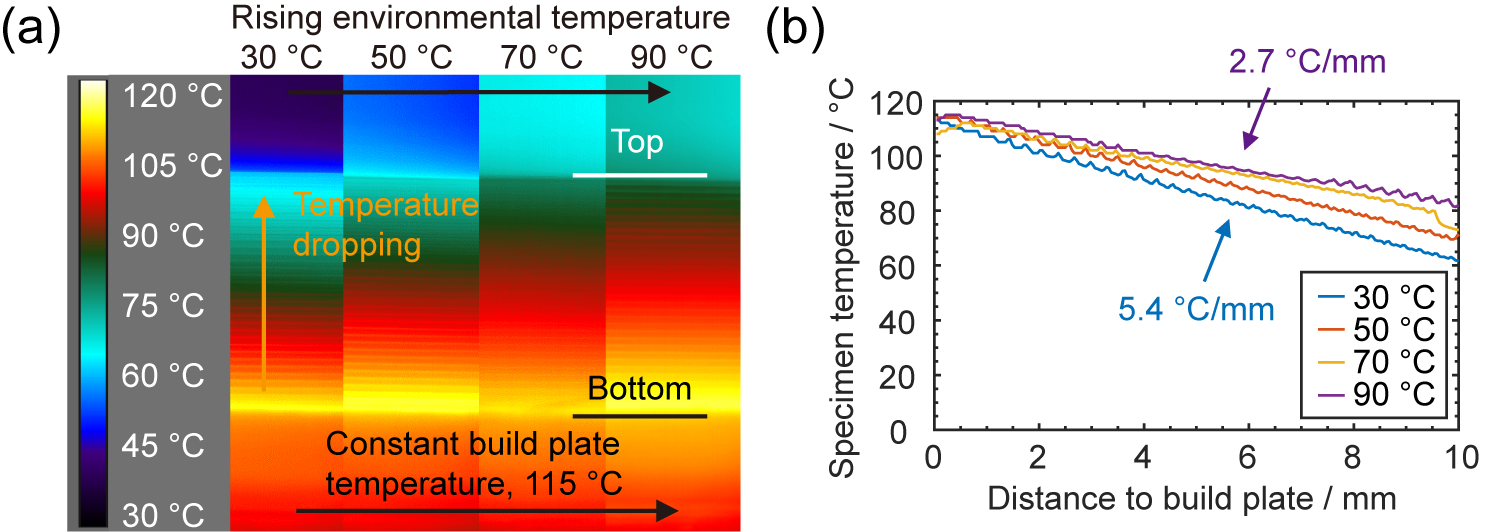

Supplement: Supplementary file 1 [file materials-13-04414-s001.zip › Fig3.tif]

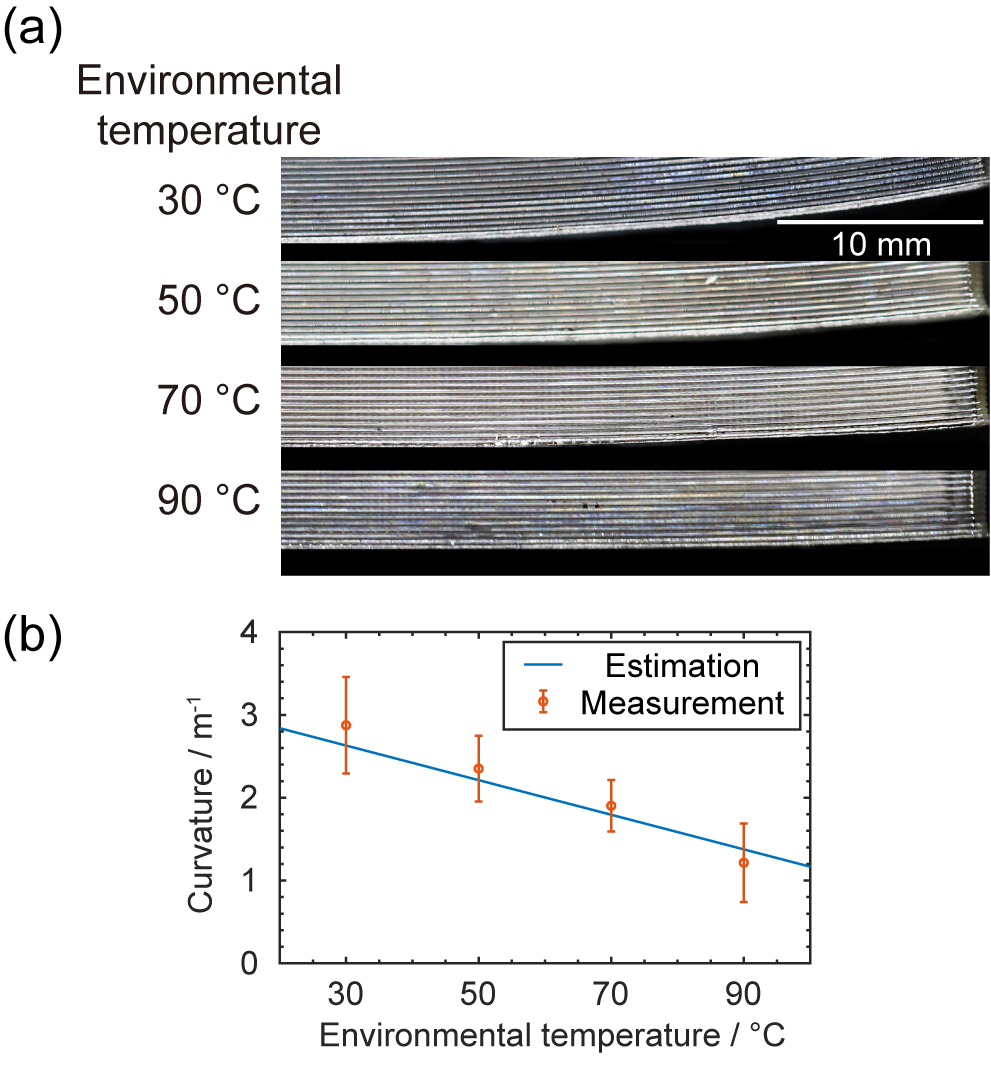

Supplement: Supplementary file 1 [file materials-13-04414-s001.zip › Fig4.tif]

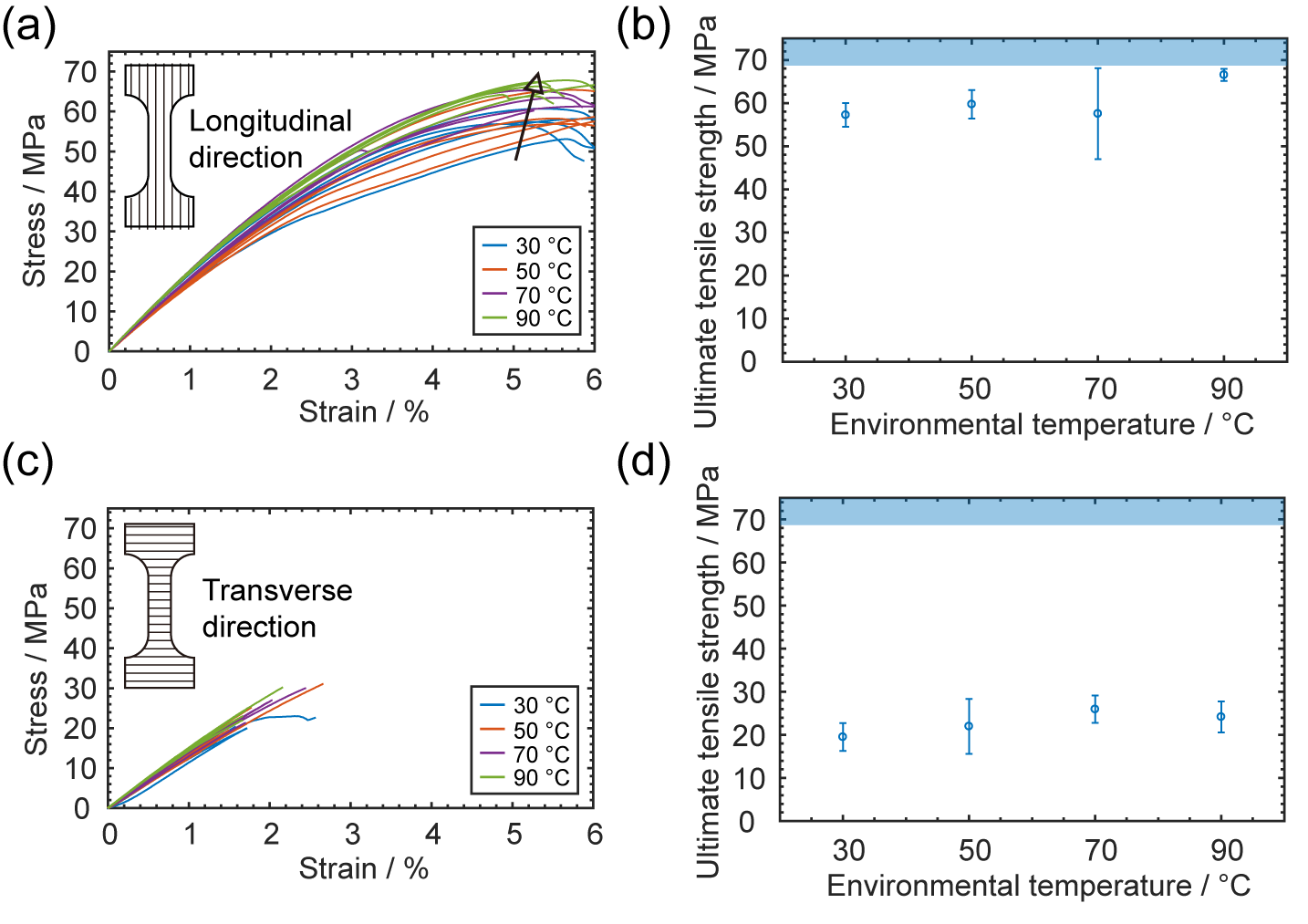

Supplement: Supplementary file 1 [file materials-13-04414-s001.zip › Fig5.tif]

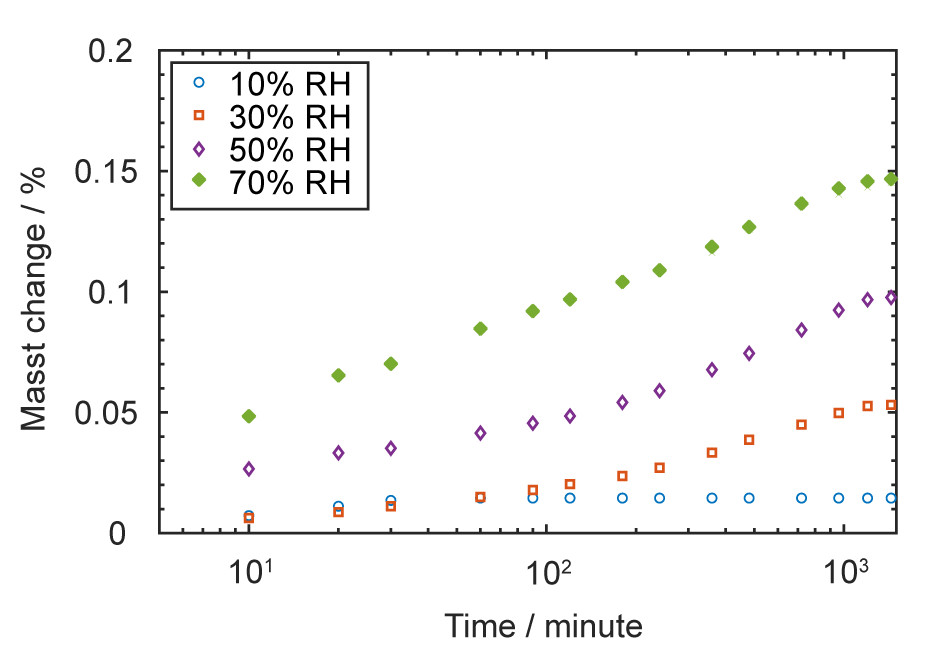

Supplement: Supplementary file 1 [file materials-13-04414-s001.zip › Fig6.tif]

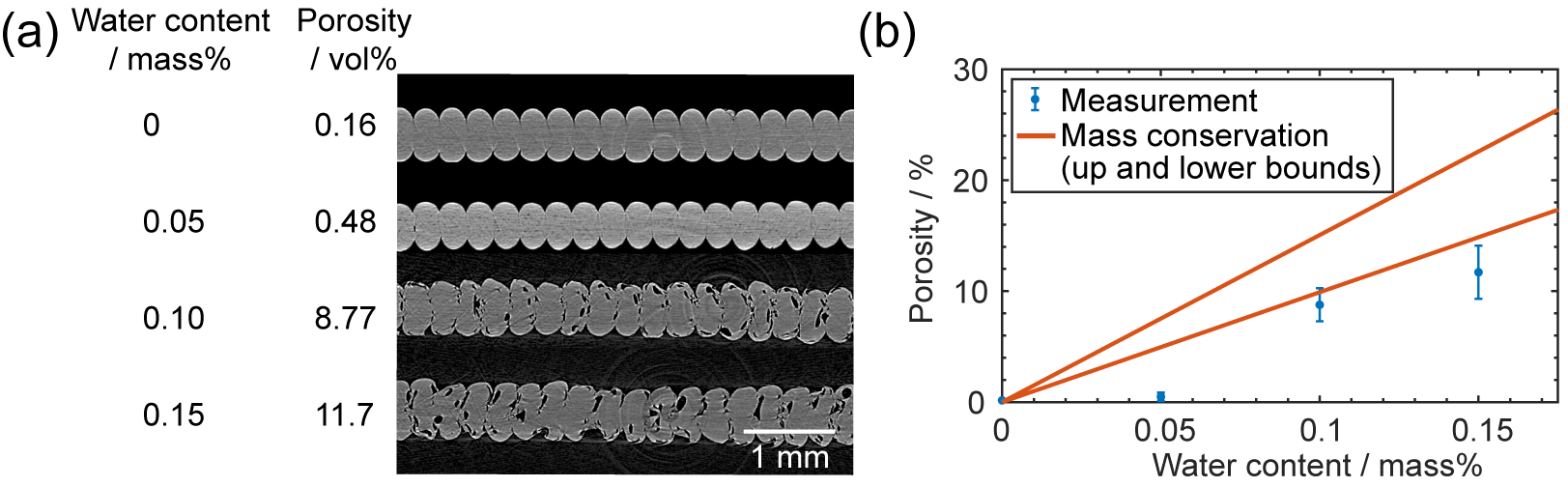

Supplement: Supplementary file 1 [file materials-13-04414-s001.zip › Fig7.tif]

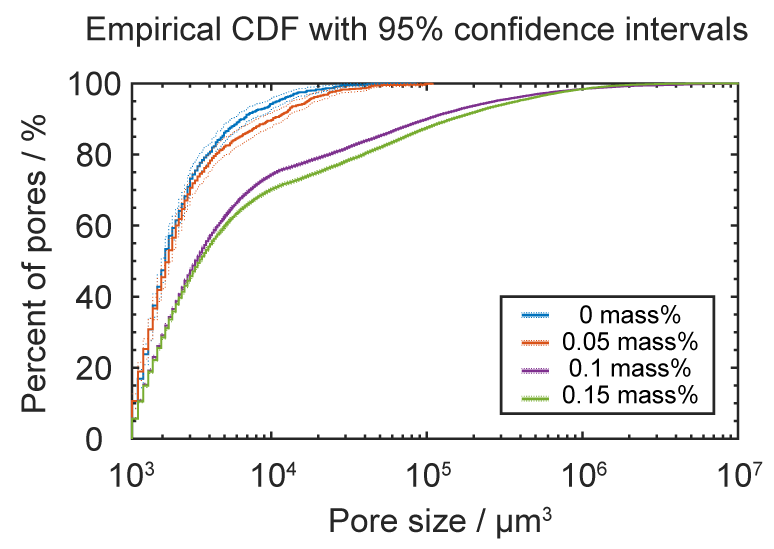

Supplement: Supplementary file 1 [file materials-13-04414-s001.zip › Fig8.tif]

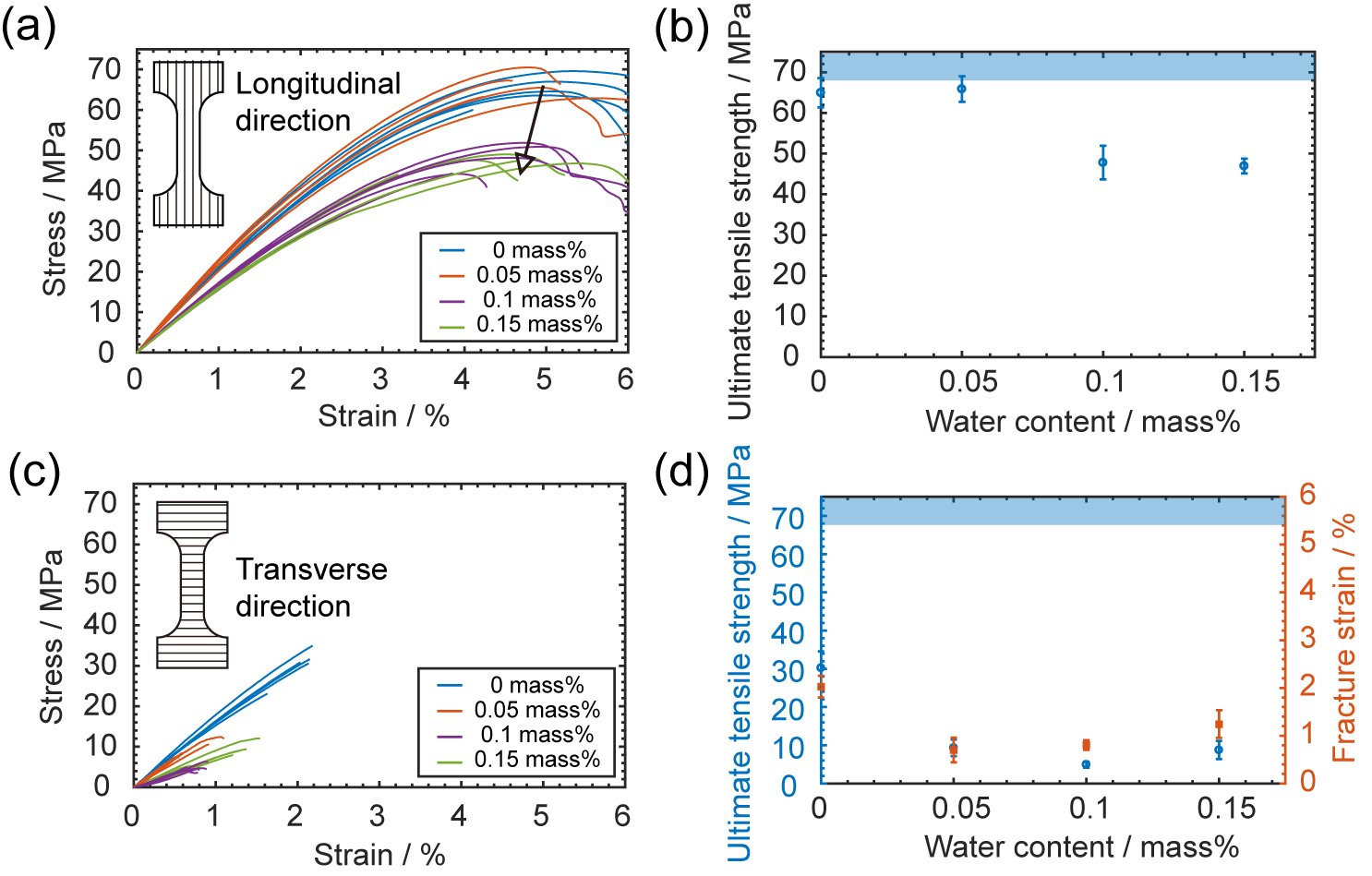

Supplement: Supplementary file 1 [file materials-13-04414-s001.zip › Fig9.tif]

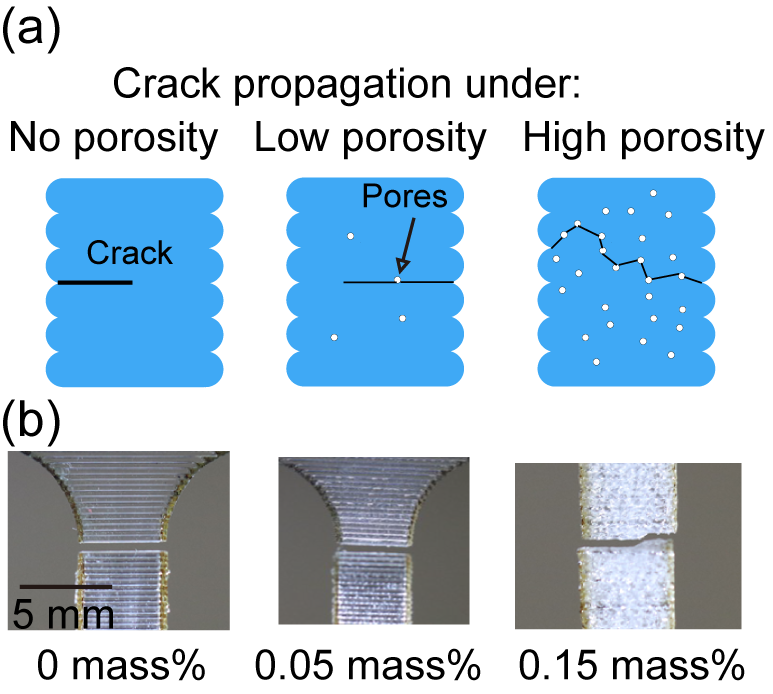

Supplement: Supplementary file 1 [file materials-13-04414-s001.zip › Fig10.tif]
